# Effects of different dietary threonine and glycine supplies in broilers fed low-protein diets

**DOI:** 10.3389/fvets.2024.1373348

**Published:** 2024-03-25

**Authors:** Patrik Strifler, Boglárka Horváth, Nikoletta Such, Károly Dublecz, László Pál

**Affiliations:** Department of Nutrition and Nutritional Physiology, Institute of Physiology and Nutrition, Hungarian University and Agriculture and Life Sciences, Keszthely, Hungary

**Keywords:** broiler, low protein diet, threonine, glycine, nitrogen

## Abstract

The reduction of crude protein (CP) content of broiler diets with balanced amino acid supply can increase the nitrogen (N) utilization efficiency and reduce ammonia emission, the risk of many health problems in birds. Feeding low protein (LP) diets without the impairment of performance traits needs the optimized dietary levels of threonine (Thr) and the non-essential amino acid (AA) glycine (Gly) and serine (Ser). However, the required concentrations and interactions of Thr and Gly + Ser, expressed as Gly equivalent (Gly_equi_), in LP diets are not fully understood. Therefore, the aim of this study was to investigate the effects of three LP (LP1–3) grower (11–24 days) and finisher (25–35 days) diets with 2% CP reduction compared to the control (C), differing in standardized ileal digestible (SID) Thr to lysine (Lys) ratio (C, LP1, LP3: 63%, LP2: 72%) and Gly_equi_ levels (C: 15.65 g/kg, LP1: 13.74 g/kg, LP2: 13.70 g/kg, LP3: 15.77). The LP treatments did not impair the performance traits of broilers. The LP2 treatment with increased SID Thr-to-Lys ratio (+9.0%) resulted in significantly higher body weight gain and a more advantageous feed conversion ratio in the whole fattening compared to the control treatment with normal CP level (*p* < 0.05). The LP3 treatment containing swine meat meal with similar Gly_equi_ levels compared to the normal CP treatment led to the most advantageous feed conversion ratio in the finisher phase and the highest nitrogen retention efficiency (*p* < 0.05). However, the LP3 treatment with a high starch-to-CP ratio negatively influenced the relative carcass weight and the ratio of abdominal fat of broilers (*p* < 0.05).

## 1 Introduction

Feeding low-protein (LP) diets to broilers has been the most effective method to lower nitrogen (N) excretion and increase N utilization efficiency, reducing the risk of wet litter problems and incidence of dysbiosis (1–3). Our current knowledge allows the reduction of up to 2% dietary crude protein (CP) in maize/soybean meal-based broiler diets in each feeding phase without performance loss and deterioration of product quality (4). This level of CP reduction can be successfully achieved by a precise adjustment of the essential amino acid (AA) supply of birds using the ‘ideal protein concept’ on a standardized ileal digestible (SID) amino acid basis (5). A wide range of free crystalline AA as feed supplements are available and can be used for this purpose.

Threonine (Thr) has been usually the third limiting AA in broiler diets, and feed grade L-Thr has been commercially available since the 1980s (6). Thr mainly serves as a substrate for the synthesis of proteins, mucin, and immunoglobulins and plays a crucial part in stress response and maintenance of gut epithelium integrity (7). The ideal ratios of essential AA should be considered even more carefully in LP diets. The ideal digestible Thr-to-lysine (Lys) ratio has been increased up to higher than 65% in diets for modern broilers, which can be further adjusted according to the target performance trait, stress level, and the challenges of the immune system (8, 9). Thr can be metabolized by either Thr aldolase or Thr dehydrogenase to glycine (Gly) in poultry, which is the precursor AA of uric acid synthesis (10). In addition, Gly can be metabolized from serine (Ser), and this reaction can be reversed (11, 12). The metabolic interconversion of Gly and Ser is continuous and unlimited, and Ser has the same effects as Gly on an equimolar basis (11). Therefore, the calculation of a dietary Gly equivalent (Gly_equi_) has been recommended using the molar equivalent of Ser being 0.7143 (13). Dietary Gly + Ser levels largely decrease when CP is reduced in vegetarian diets, and these AAs become the first growth-limiting non-essential AA when the CP content of diets is below 19% during 7 to 21 days of age and below 17% during 21 to 35 days of age (14, 15). The interrelationship of dietary Thr and Gly_equi_ is quite complex and has been reported by many studies (16–21). Dietary Thr levels higher than recommended may reduce the requirement of dietary Gly_equi_ to achieve certain response levels of performance traits (22). This so-called sparing or replacement effect can be partly attributed to the conversion of Thr to Gly (23). Furthermore, the increasing dietary levels of Thr may reduce the catabolism of other AAs, thereby reducing the need for Gly_equi_ for uric acid formation (23). On the other hand, Gly supplementation decreases the activity of the enzymes Thr aldolase and Thr dehydrogenase, which can lead to a decreased degradation of Thr and an increased availability of Thr for physiological needs (19).

The interaction between Thr and Gly concerning broiler performance has been studied mostly during the first 3 weeks of broiler life. The interactions between Thr and Gly_equi_ from 7 to 21 days were quantified by Siegert et al. (22). They found that the increasing dietary Thr reduced the Gly_equi_ required to achieve certain BWG and FCR responses. The effects of an additional 0.2% or 0.4% Gly were dependent on the CP level of the diet fed from day 0 to day 21 in the study by Waldroup et al. (16). When Gly was added to diets with 16% or 18% CP (1.30 or 1.62% Gly + Ser), the BW of birds at 21 days of age was significantly improved. However, the addition of Gly to diets with 20, 22%, or 24% CP (1.86, 2.08, and 2.28% Gly + Ser) showed no or little benefit. The authors did not observe any performance improvement of an additional 0.2% or 0.4% Thr (0.80–0.98% Thr in basal diets) or interaction between Gly and Thr. Ospina-Rojas et al. (18) investigated an LP diet with 19% CP fed from day 0 to day 21 with two Thr levels (0.93 and 1.07% Thr) and four concentrations of Gly + Ser (1.84 to 2.26%). The increasing supplemental Gly + Ser improved the FCR of birds in a quadratic manner at 0.93% Thr level, but it had no significant effect on the FCR at 1.07% Thr concentration. The research focusing on the interaction between Thr and Gly in the grower-finisher phase of fattening is more limited and needs further precise quantification. The FCR of broilers from 21 to 35 days showed a stronger response to Gly when the digestible Thr-to-Lys ratio of diets was 65% compared to the 72% digestible Thr-to-Lys ratio (19). Broilers fed a diet with a lower digestible (Gly + Ser)-to-Lys level showed a stronger response of FCR to dietary Thr level (135% vs. 149%) (19). Corzo et al. (17) also observed similar interactions for BWG of birds from 21 to 42 days of age. In the study by Star et al. (21), the BWG of broilers from 7 to 28 days of age only responded when both dietary Gly and Thr levels were very low. In addition to their respective concentrations in diets, the effects of dietary Thr and Gly are also related to the dietary level of Met+Cys, choline, Arg, guanidino acetic acid, and creatine, as reviewed by Siegert and Rodehutscord (23).

Most of the studies focusing on the Thr-Gly interactions applied only one LP diet and crystalline Gly and Thr supplementation (17–19). The objective of this study was to use practically formulated diets with both normal and low CP content without crystalline Gly, which is only allowed as a flavoring agent in the EU. To increase the Gly concentration of LP diets, swine meat meal rich in Gly was used in our experiment. Furthermore, the present study focuses not only on the Thr and Gly interactions concerning broiler performance but on the carcass characteristics, meat quality, efficiency of nitrogen retention, and nitrogen forms of excreta using practically formulated LP diets suitable in the EU.

## 2 Materials and methods

### 2.1 Experimental animals and treatments

A floor pen trial was carried out at the experimental farm of the Institute of Physiology and Nutrition, Georgikon Campus, Hungarian University of Agriculture and Life Sciences (Keszthely, Hungary). A total of 576 1-day-old male broiler chickens (Ross 308) were purchased from a local hatchery (Gallus Ltd., Devecser, Hungary) and divided randomly into 24 floor pens at a stocking rate of 24 birds per pen (14 bird/m^2^). Animals were vaccinated against infectious bronchitis (CEVAC BRON), Newcastle disease (CEVAC VITAPEST), and infectious bursal disease (CEVAC TRANSMUNE) in the hatchery using vaccines produced by Ceva (Ceva Santé Animale, France). Chopped wheat straw was used as litter material. The animals were provided *ad libitum* water and feed during the entire duration of the experiment. The climatic conditions and light program, based on the breeder’s guidelines, were computer-controlled and identical for all pens. The room temperature was set to 34°C on day 0 and reduced gradually to 24°C at 18 days of age. The light intensity was 30 lux in the first week and 10 lux thereafter, with a constant day length of 23 h from day 0 to day 7 and 20 h light and 4 h dark period thereafter.

Three dietary phases were used during the 35-day-long experiment: starter (from 0 to 10 days), grower (from 11 to 24 days), and finisher (from 25 to 35 days). All birds were fed the same starter phase diet, and 4 dietary treatments consisting of 6 replicates with 24 birds in each were established and experimental diets were fed in the grower and finisher phases in the pelleted form. The design of the experiment is described in Table 1. Diets of the control C treatment were formulated in line with the breeder’s recommendations for Ross 308 (Aviagen, Newbridge, United Kingdom) and adequate levels of CP and SID essential AA. Low protein (LP) diets, LP1, LP2, and LP3, contained 2.0% less crude protein than diet C with control CP level in each dietary phase. Dietary treatments were different in SID Thr level and SID Thr-to-Lys ratio as well as SID (Gly + Ser)-to-Lys ratio and the Gly_equi_. The composition of experimental diets is shown in Table 2, while the calculated and measured nutrient content of experimental diets can be seen in Table 3. The experimental LP diets were isocaloric with diet C. The increased Gly_equi_ in the LP3 diet was achieved by the partial replacement of soybean meal with swine meat meal rich in Gly. Diets were formulated based on standardized ileal digestible (SID) AAs in accordance with the ideal protein concept. LP diets were supplemented with six feed-grade crystalline essential AAs (Lys, Met, Val, Thr, Arg, and Ile) to meet the calculated concentrations of SID AAs in the C diets except for the SID Thr level of the LP2 diet. All diets contained phytase and xylanase enzymes, but no amino acid-releasing impact of these enzymes was considered in feed formulations.

**Table 1:** Experimental design.

Treatments^a^	Grower diets (11–24 days)						Finisher diets (25–35 days)					
	CP (%)	SID Thr (%)	SID Thr-to-Lys ratio (%)	Gly + Ser (%)	SID (Gly + Ser) to Lys ratio (%)	Gly_equi_ (g/kg)	CP (%)	SID Th (%)	SID Thr-to-Lys ratio (%)	Gly + Ser (%)	SID (Gly + Ser) to Lys ratio (%)	Gly_equi_ (g/kg)
C	21.00	0.74	63	1.85	137	15.65	19.00	0.65	64	1.68	143	14.19
LP1	19.00	0.73	63	1.62	121	13.74	17.00	0.65	64	1.45	124	12.30
LP2	19.00	0.84	72	1.62	121	13.70	17.00	0.74	73	1.45	124	12.26
LP3	19.00	0.73	63	1.81	130	15.77	17.00	0.65	64	1.57	130	13.55

a C - control diet; LP1 – soybean meal-based diet with reduced crude protein levels (−2%); LP2 – soybean meal-based diet with reduced crude protein levels (−2%) and higher crystalline L-Threonine supplementation; LP3 – diet with reduced crude protein levels (−2%) in which soybean meal partially replaced with swine meat meal as protein source; CP – crude protein; Gly_equi_ - Gly equivalent (g/kg feed) = glycine (g/kg) + [0.7143 x serine (g/kg)].

**Table 2:** Composition of experimental diets (%).

Ingredients	Starter (0–10 days)	Grower (11–24 days)				Finisher (25–35 days)			
		C	LP1	LP2	LP3	C	LP1	LP2	LP3
Maize	39.13	42.48	49.30	49.30	57.03	47.95	54.97	54.98	60.34
Wheat	10.00	10.00	10.00	10.00	10.00	10.00	10.00	10.00	10.00
Soybean meal extr.	40.70	37.40	30.50	30.39	20.50	32.10	25.20	25.10	18.20
Swine meat meal	0.00	0.00	0.00	0.00	6.00	0.00	0.00	0.00	4.00
Sunflower oil	5.10	6.00	5.30	5.30	3.10	6.40	5.40	5.40	4.00
Limestone	1.65	1.39	1.39	1.39	0.60	1.20	1.23	1.23	0.69
MCP	1.32	1.10	1.10	1.10	0.05	0.89	0.90	0.90	0.20
L-Lysine (Biolys)	0.41	0.27	0.56	0.56	0.69	0.21	0.50	0.50	0.61
DL-Methionine	0.40	0.32	0.37	0.37	0.40	0.29	0.34	0.34	0.37
L-Valine	0.10	0.06	0.16	0.16	0.21	0.06	0.17	0.17	0.21
L-Threonine	0.14	0.08	0.16	0.27	0.21	0.06	0.15	0.24	0.18
L-Arginine	0.03	0.00	0.15	0.15	0.21	0.00	0.19	0.19	0.25
L-Isoleucine	0.03	0.01	0.12	0.12	0.21	0.01	0.12	0.12	0.19
Salt	0.30	0.30	0.30	0.30	0.20	0.30	0.30	0.30	0.23
Sodium bicarbonate	0.10	0.10	0.10	0.10	0.10	0.10	0.10	0.10	0.10
Premix^a^	0.50	0.40	0.40	0.40	0.40	0.40	0.40	0.40	0.40
Phytase^b^	0.01	0.01	0.01	0.01	0.01	0.01	0.01	0.01	0.01
NSP enzyme^c^	0.02	0.02	0.02	0.02	0.02	0.02	0.02	0.02	0.02
Coccidiostat^d^	0.06	0.06	0.06	0.06	0.06	0.00	0.00	0.00	0.00

C - control diet; LP1 – soybean meal-based diet with reduced crude protein levels (−2%); LP2 – soybean meal-based diet with reduced crude protein levels (−2%) and higher crystalline L-Threonine supplementation; LP3 – diet with reduced crude protein levels (−2%) in which soybean meal partially replaced with swine meat meal as a protein source.

a Premix was supplied by UBM Ltd. (Pilisvörösvár, Hungary). The active ingredients contained in the premix were as follows (per kg of diet): Starter and grower premixes - retinyl acetate - 5.0 mg, cholecalciferol - 130 g, dl-alpha-tocopherol-acetate - 91 mg, menadione - 2.2 mg, thiamine - 4.5 mg, riboflavin - 10.5 mg, pyridoxin HCl - 7.5 mg, cyanocobalamin - 80 g, niacin - 41.5 mg, pantothenic acid - 15 mg, folic acid - 1.3 mg, biotin - 150 g, betaine - 670 mg, monensin-Na - 110 mg (only grower), narasin - 50 mg (only starter), nicarbazin - 50 mg (only starter), antioxidant - 25 mg, Zn (as ZnSO_4_H_2_O) - 125 mg, Cu (as CuSO_4_5H_2_O) - 20 mg, Fe (as FeSO_4_H_2_O) - 75 mg, Mn (as MnO) - 125 mg, I (as KI) - 1.35 mg, Se (as Na_2_SeO_3_) - 270 g; Finisher premix - retinyl acetate - 3.4 mg, cholecalciferol - 97 g, dl-alpha-tocopherol-acetate - 45.5 mg, menadione - 2.7 mg, thiamin - 1.9 mg, riboflavin - 5.0 mg, pyridoxin HCl - 3.2 mg, cyanoco-balamin - 19 g, niacin - 28.5 mg, pantothenic acid - 10 mg, folic acid - 1.3 mg, biotin - 140 g, L-ascorbic acid - 40 mg, betaine - 193 mg, antioxidant - 25 mg, Zn (as ZnSO_4_H_2_O) - 96 mg, Cu - 9.6 mg, Fe (as FeSO_4_H_2_O) - 29 mg, Mn (as MnO) - 29 mg, I (as KI) - 1.2 mg, Se (as Na_2_SeO_3_) - 350 g.

b Axtra® Phy 5,000 TPT phytase 500 FTU (Danisco Animal Nutrition & Health, USA).

c Danisco Xylanase 8,000 G (Danisco Animal Nutrition & Health, USA).

d Maxiban® G160 premix (Elanco Animal Health, Australia).

**Table 3:** Calculated and measured the nutrient content of the experimental diets (%).

Calculated nutrients	Starter (0–10 days)	Grower (11–24 days)				Finisher (25–35 days)			
		C	LP1	LP2	LP3	C	LP1	LP2	LP3
Crude protein	22.50	21.00	19.00	19.00	19.00	19.00	17.00	17.00	17.00
AMEn (MJ/kg)	12.55	13.05	13.08	13.07	13.12	13.46	13.41	13.40	13.46
Starch	33.16	35.02	38.80	38.80	42.94	38.06	41.97	41.97	44.84
Crude fat	7.29	8.24	7.64	7.60	6.12	8.72	7.83	7.83	6.89
SID Lysine	1.30	1.15	1.16	1.16	1.15	1.01	1.01	1.01	1.01
SID Methionine	0.70	0.60	0.62	0.62	0.65	0.55	0.57	0.57	0.60
SID Met+Cys	0.98	0.88	0.87	0.87	0.87	0.81	0.80	0.80	0.81
SID Arginine	1.40	1.29	1.24	1.24	1.23	1.14	1.14	1.14	1.15
SID Threonine	0.83	0.74	0.73	0.84	0.73	0.65	0.65	0.74	0.65
SID Valine	0.97	0.88	0.87	0.87	0.87	0.80	0.80	0.80	0.81
SID Isoleucine	0.85	0.79	0.78	0.78	0.78	0.70	0.70	0.70	0.71
SID Glycine	0.76	0.72	0.63	0.63	0.80	0.65	0.56	0.56	0.67
SID Serine	0.92	0.87	0.77	0.76	0.70	0.79	0.69	0.69	0.64
Ca	1.06	0.92	0.91	0.91	0.92	0.80	0.80	0.80	0.80
P_available_	0.51	0.46	0.45	0.45	0.46	0.40	0.40	0.40	0.40
Gly + Ser	1.96	1.85	1.62	1.62	1.81	1.68	1.45	1.45	1.57
SID Thr-to-Lys ratio	0.64	0.63	0.63	0.72	0.63	0.64	0.64	0.73	0.64
Gly_equi_ (g/kg)^a^	16.56	15.65	13.74	13.70	15.77	14.19	12.30	12.26	13.55
AMEn-to-CP ratio^b^	0.56	0.62	0.69	0.68	0.69	0.71	0.78	0.78	0.79
Starch-to-CP ratio^c^	1.47	1.67	2.03	2.03	2.25	2.00	2.45	2.44	2.63
Measured nutrients									
Dry matter	89.07	89.16	89.37	89.26	89.30	89.72	89.64	89.59	89.69
Crude protein	22.27	21.25	19.20	19.17	19.08	19.23	17.14	17.18	17.05
Lysine	1.43	1.27	1.26	1.26	1.27	1.11	1.09	1.09	1.13
Methionine	0.72	0.63	0.65	0.66	0.68	0.58	0.59	0.60	0.62
Met+Cys	1.07	0.96	0.94	0.93	0.95	0.88	0.87	0.89	0.88
Arginine	1.53	1.40	1.35	1.36	1.37	1.24	1.25	1.26	1.27
Glycine	0.88	0.83	0.72	0.74	1.05	0.74	0.65	0.63	0.85
Serine	1.07	1.01	0.88	0.87	0.82	0.91	0.76	0.77	0.73
Gly + Ser	1.95	1.84	1.59	1.61	1.87	1.65	1.41	1.40	1.58
Threonine	0.97	0.86	0.84	0.94	0.85	0.76	0.73	0.75	0.76
Valine	1.08	0.99	0.96	0.97	0.98	0.89	0.88	0.87	0.89
Isoleucine	0.97	0.89	0.87	0.86	0.87	0.79	0.77	0.78	0.79
Ca	1.06	0.92	0.90	0.94	0.89	0.85	0.84	0.82	0.85
P	0.61	0.64	0.58	0.58	0.58	0.54	0.52	0.52	0.52

C - control diet; LP1 – soybean meal-based diet with reduced crude protein levels (−2%); LP2 – soybean meal-based diet with reduced crude protein levels (−2%) and higher crystalline L-Threonine supplementation; LP3 – diet with reduced crude protein levels (−2%) in which soybean meal partially replaced with swine meat meal as protein source.

a Gly equivalent (g/kg feed) = glycine (g/kg) + [0.7143 x serine (g/kg)].

b Ratio of dietary AMEn and crude protein concentration.

c Ratio of dietary starch and crude protein concentration.

### 2.2 Measurements

The body weight (BW) of broilers was measured individually at the start of the trial and the end of each dietary phase, and the mean BW was calculated for each pen. Feed intake (FI) of broilers was recorded per pen at the end of each dietary phase. Body weight gain (BWG) and feed conversion ratio (FCR) were calculated per pen at the end of each phase and for the whole trial period. Mortality and the weight of dead birds were registered daily during the whole trial. At day 35, two chickens with average BW from each pen (12 birds per treatment) were randomly selected and transferred to balance cages, where chickens consumed the same finisher diets but supplemented with 0.5% TiO_2_ as an indigestible internal marker. After 5 days adaptation period, representative excreta samples were collected from each bird daily for 2\u00B0consecutive days (days 41 and 42). The samples of 12 birds per treatment were pooled, mixed thoroughly, frozen, and stored at −20°C until further analyses. Before the analyses, excreta was homogenized properly, and then the dry matter content, total-N, ammonium-N (NH_4_^+^-N), and uric acid-N contents were determined. The dry matter content of excreta samples was measured in an exicator (100°C for 24 h). The total N of excreta was determined according to the Kjeldahl method with Foss-Kjeltec 8,400 Analyzer Unit (Nils Foss Allé 1, DK-3400 Hilleroed, Denmark), the ammonium-N by the method of Peters (24), and the uric acid-N as described by Marquardt (25). All N parameters were adjusted on a dry matter basis. The sum of NH_4_^+^-N and uric acid-N was considered as urinary N content (26). Feed samples were analyzed for dry matter (ISO 6496), crude protein (ISO 5983-1:2005), phosphorus (ISO 6491), calcium (ISO 6896) content, and amino acid composition (ISO 13903:2005) using methods of International Organization for Standardization (ISO). The TiO_2_\u00B0concentration of experimental diets and excreta samples was determined using a UV-spectroscopy assay (27). Nitrogen retention was calculated using the following equation (28): Apparent nitrogen retention = 1 – [([TiO_2_] diet/[TiO_2_] excreta) × ([nitrogen] excreta/[nitrogen] diet)]. At the end of the experiment, two birds per pen (12 birds per treatment) representing the average BW of the pen were selected to be slaughtered by cervical dislocation. After evisceration, carcass composition (% of carcass weight, % of breast meat, % of thigh weight, % of abdominal fat) and breast meat quality were determined. The pH of the breast muscle, *Pectoralis major* (*P. major*), was measured immediately after slaughtering (pH_0h_) and after 24 h storage at 4°C (pH_u_) with a portable pH meter (Testo 205; Testo Ltd., Hungary) by inserting a glass electrode directly in the thickest part of the muscle. The water-holding capacity of meat was estimated by measuring drip loss of the raw meat: the *P. major* muscle was weighed immediately after slaughter and placed in a plastic bag, hung from a hook, and stored at 4°C for 24 h. After hanging, the sample was wiped with an absorbent paper and weighed again. The difference in weight corresponding to the drip loss was expressed as the percentage of the initial muscle weight (29).

### 2.3 Statistical analysis

The averages of examined parameters were analyzed in a completely randomized design using a one-way analysis of variance (ANOVA) with dietary treatments as the main effects. For performance results (BW, BWG, FI, and FCR), the pen was the experimental unit, whereas for other variables, the individual bird was the experimental unit. When the F-test revealed a significant treatment effect, the significant differences between groups were tested by the Tukey HSD test. All statistical analyses were carried out using the software package SPSS 22.0 for Windows (IBM Corp., Armonk, NY, United States). Statistical significance has been declared at *p* < 0.05.

## 3 Results

There were no remarkable differences between measured and calculated values of dietary total AA (Table 3). The effect of dietary treatments on the total intake of the balanced SID essential AA without Thr and the intake of SID Thr and Gly + Ser of broilers in the grower and finisher phases is presented in Table 4. The total intake of the balanced SID essential AA without Thr was not significantly different among treatment groups. In addition, the results show that the calculated differences between SID Thr and Gly + Ser levels in the dietary treatments resulted in significant differences in SID Thr and Gly + Ser intake of broilers.

**Table 4:** Intake of SID EAA^1^, SID Thr, and Gly + Ser of broilers during the feeding phases (g/bird, means of 6 pens per treatment; *n* = 6).

Treatment^3^	SID EAA			SID Thr			Gly + Ser		
	Grower	Finisher	G + F^2^	Grower	Finisher	G + F	Grower	Finisher	G + F
C	63.55	82.42	145.98	9.43^b^	12.01^b^	21.44^b^	23.43^a^	30.49^a^	53.93^a^
LP1	63.81	85.27	149.08	9.47^b^	12.46^b^	21.92^b^	20.62^b^	27.02^b^	47.64^b^
LP2	64.26	83.18	147.44	10.97^a^	13.83^a^	24.80^a^	21.02^b^	26.17^b^	47.20^b^
LP3	62.71	84.53	147.24	9.34^b^	12.24^b^	21.58^b^	23.94^a^	29.75^a^	53.68^a^
Pooled SEM	0.52	0.74	1.07	0.16	0.18	0.32	0.35	0.45	0.66
*p*-value	NS^4^	NS	NS	<0.001	<0.001	<0.001	<0.001	<0.001	<0.001

^1^Total intake of balanced SID essential amino acids without Thr: Lys + Met+Cys + Arg + Val + Ile; ^2^Grower+Finisher; ^3^C - control diet; LP1 – soybean meal-based diet with reduced crude protein levels (−2%); LP2 – soybean meal based diet with reduced crude protein levels (−2%) and higher crystalline L-Threonine supplementation; LP3 – diet with reduced crude protein levels (−2%) in which soybean meal partially replaced with swine meat meal as a protein source; ^4^NS – non-significant (*p* > 0.05); ^a,b^Means with different superscripts in the same column are significantly different (*p* < 0.05).

The results of production parameters are shown in Table 5. The performance parameters were not significantly influenced by the same starter diet fed in all pens when the feeding of experimental diets started on day 10. There were no significant differences between the BW of the treatment groups at the end of the grower phase, while in the finisher phase, the broilers fed the LP2 diet showed a significantly higher BW than the broilers consuming the C diet (*p* < 0.05). The BWG of birds in the grower, finisher, and whole trial period was significantly affected by the experimental diets (*p* < 0.05). In the grower phase, the feeding of LP diets did not lead to significantly different BWG compared to the C treatment. However, there was a significant difference between the LP2 and LP3 groups: the increased SID Thr-to-Lys level of the LP2 diet resulted in a higher BWG of birds than the LP3 diet with higher Gly_equi_ (*p* < 0.05). The BWG of broilers in the LP1 and LP3 groups was significantly higher than that in the C group in the finisher phase (*p* < 0.05). As for the whole experiment, only the broilers fed the LP2 diet achieved a significantly higher BWG than the birds in the C treatment (*p* < 0.05). In contrast to BW and BWG data, no significant differences were found in the FI of experimental animals among the treatments in any phases of the experiment. The FCR of broilers was significantly influenced by dietary treatments in both the grower and finisher phases as well as during the whole fattening (*p* < 0.05). In the grower phase, the LP diets did not lead to significantly different FCR values than the C diet. As for FCR in LP treatments, the same significant difference was seen between LP2 and LP3 as it was observed in the case of BWG (*p* < 0.05). Experimental animals fed the LP3 diet showed better FCR than the control birds consuming the C diet in the finisher phase (*p* < 0.05). The FCR value calculated for the whole study in the LP2 group exceeded the FCR in the C group but did not differ significantly from the two other LP treatments.

**Table 5:** Performance parameters of birds in the starter, grower, and finisher phases and in the whole experiment (mean ± SEM; *n* = 6 pens per treatment).

	Treatment^1^	0 d	10 d	24 d	35 d
Body weight (g/bird)	C	47.9 ± 0.1	269.9 ± 8.0	1247.8 ± 14.5	2479.9 ± 25.6^b^
	LP1	47.9 ± 0.1	271.4 ± 9.2	1266.3 ± 22.9	2617.1 ± 46.2^ab^
	LP2	48.2 ± 0.2	273.2 ± 7.2	1305.2 ± 29.1	2633.3 ± 37.9^a^
	LP3	48.3 ± 0.1	289.5 ± 3.7	1226.7 ± 25.4	2579.2 ± 30.4^ab^
	*p*-value	NS^2^	NS	NS	0.029
		Starter (0–10 days)	Grower (11–24 days)	Finisher (25–35 days)	Whole trial (0–35 days)
Body weight gain (g/bird)	C	222.0 ± 8.0	977.9 ± 10.3^ab^	1232.1 ± 26.8^b^	2432.0 ± 25.6^b^
	LP1	223.5 ± 9.3	994.9 ± 19.2^ab^	1350.8 ± 35.8^a^	2569.2 ± 46.1^ab^
	LP2	225.0 ± 7.1	1031.9 ± 23.9^a^	1328.1 ± 22.4^ab^	2585.0 ± 37.9^a^
	LP3	244.7 ± 1.2	937.1 ± 24.2^b^	1352.5 ± 23.8^a^	2530.9 ± 30.3^ab^
	*p*-value	NS	0.027	0.018	0.030
Feed intake (g/bird)	C	270.8 ± 3.7	1273.6 ± 24.6	1848.1 ± 31.4	3351.0 ± 16.8
	LP1	276.7 ± 4.3	1297.1 ± 18.0	1916.1 ± 48.6	3489.9 ± 59.7
	LP2	272.9 ± 5.5	1306.1 ± 21.4	1869.2 ± 20.5	3419.1 ± 9.6
	LP3	283.3 ± 0.6	1279.9 ± 23.7	1882.6 ± 29.3	3447.7 ± 50.5
	*p*-value	NS	NS	NS	NS
Feed conversion ratio (kg/kg)	C	1.23 ± 0.03	1.30 ± 0.02^ab^	1.50 ± 0.02^b^	1.40 ± 0.01^a^
	LP1	1.25 ± 0.05	1.30 ± 0.01^ab^	1.42 ± 0.03^ab^	1.36 ± 0.01^ab^
	LP2	1.22 ± 0.03	1.27 ± 0.01^a^	1.43 ± 0.01^ab^	1.33 ± 0.01^b^
	LP3	1.16 ± 0.01	1.37 ± 0.03^b^	1.39 ± 0.02^a^	1.36 ± 0.01^ab^
	*p*-value	NS	0.016	0.013	0.026

^1^C - control diet; LP1 – soybean meal-based diet with reduced crude protein levels (−2%); LP2 – soybean meal-based diet with reduced crude protein levels (−2%) and higher crystalline L-Threonine supplementation; LP3 – diet with reduced crude protein levels (−2%) in which soybean meal partially replaced with swine meat meal as a protein source.; ^2^NS – non-significant (*p* > 0.05); ^a,b^Means with different superscripts in the same column are significantly different (*p* < 0.05).

The dietary treatments significantly affected the relative carcass weight and abdominal fad pad (*p* < 0.05), while the relative breast meat yield and thigh weight were not significantly influenced by experimental feeding (Table 6). The relative carcass weight of broilers in the LP1 and LP2 groups was not different from the C group. However, this trait in the LP3 treatment was significantly lower than in the C and LP2 treatments. Furthermore, the feeding of the LP3 diet resulted in a higher relative abdominal fat pad compared to the C diet (*p* < 0.05). The dietary treatments did not significantly influence the drip loss of breast meat or the pH of the breast meat fillet measured either immediately after slaughter or after 24 h (*p* > 0.05; Table 7).

**Table 6:** Carcass weight and composition^1^ (%, mean ± SEM; n = 12 broilers per treatment).

Treatment^2^	Carcass weight	Breast meat yield	Thigh weight	Abdominal fat
C	65.67 ± 0.45^a^	22.37 ± 0.35	19.21 ± 0.29	0.68 ± 0.09^b^
LP1	65.18 ± 0.42^ab^	21.20 ± 0.50	19.00 ± 0.20	0.98 ± 0.09^ab^
LP2	66.25 ± 0.25^a^	22.25 ± 0.34	19.15 ± 0.20	0.87 ± 0.08^ab^
LP3	64.06 ± 0.32^b^	20.92 ± 0.45	18.92 ± 0.19	1.04 ± 0.09^a^
*p*-value	<0.001	NS^3^	NS	0.032

^1^Values expressed as a percentage of live BW; ^2^C - control diet; LP1 – soybean meal-based diet with reduced crude protein levels (−2%); LP2 – soybean meal-based diet with reduced crude protein levels (−2%) and higher crystalline L-Threonine supplementation; LP3 – diet with reduced crude protein levels (−2%) in which soybean meal partially replaced with swine meat meal as a protein source; ^3^NS – non-significant (*p* > 0.05); ^a,b^Means with different superscripts in the same column are significantly different (*p* < 0.05).

**Table 7:** Breast meat quality parameters (mean ± SEM; *n* = 12 broilers per treatment).

Treatment^1^	pH_0h_^2^	pH_u_^3^	Drip loss (%)
C	6.49 ± 0.06	5.76 ± 0.02	1.08 ± 0.06
LP1	6.47 ± 0.04	5.82 ± 0.02	1.07 ± 0.09
LP2	6.56 ± 0.05	5.85 ± 0.03	1.04 ± 0.05
LP3	6.59 ± 0.04	5.79 ± 0.02	1.10 ± 0.06
p-value	NS^4^	NS	NS

^1^C - control diet; LP1 – soybean meal-based diet with reduced crude protein levels (−2%); LP2 – soybean meal-based diet with reduced crude protein levels (−2%) and higher crystalline L-Threonine supplementation; LP3 – diet with reduced crude protein levels (−2%) in which soybean meal partially replaced with swine meat meal as a protein source; ^2^pH_0h_ = pH measured immediately after slaughter; ^3^pH_u_ = pH measured after 24 h storage at 4°C; ^4^NS – non-significant (*p* > 0.05).

**Figure 1:**
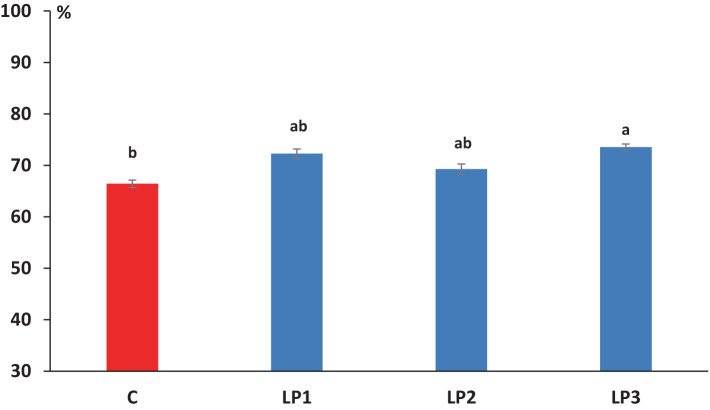
Effect of dietary treatments on the efficiency of dietary N retention (mean + SEM; *n* = 12 broilers per treatment). ^a,b^Means with different superscripts are significantly different; C - control diet; LP1 – soybean meal-based diet with reduced crude protein levels (−2%); LP2 – soybean meal-based diet with reduced crude protein levels (−2%) and higher crystalline L-Threonine supplementation; LP3 – diet with reduced crude protein levels (−2%) in which soybean meal partially replaced with swine meat meal as protein source.

The effect of dietary treatments on the nitrogen retention efficiency of broilers was significant (66.4, 72.3, 69.3, and 73.5% in the C, LP1, LP2, and LP3 groups, respectively; Figure 1; *p* < 0.05). The experimental animals of treatment LP3 achieved significantly higher nitrogen retention efficiency than the birds in the C group (*p* < 0.05). The mean dry matter content of excreta was 21.0, 23.9, 24.4, and 22.4% in the C, LP1, LP2, and LP3 treatment groups, respectively, and showed only the tendency of increase in the LP groups compared to the C treatment (*p* = 0.109). The dietary treatments did not significantly influence the concentration of fecal-N, uric acid-N, NH_4_^+^-N, urinary-N, and total-N in the excreta of broiler chickens. The ratio of urinary-N within the total-N in excreta was 43.1, 42.7, 42.3, and 42.7% in the C, LP1, LP2, and LP3 groups, respectively, and these values were not affected by dietary treatments (see Table 8).

**Table 8:** The concentration of N-forms in broiler excreta (mean ± SEM; *n* = 12 broilers per treatment).

Treatment^1^	Fecal-N	NH_4_^+^-N	Uric acid-N	Urinary-N^2^	Total-N
	mg/g dry matter				
C	21.87 ± 0.71	3.91 ± 0.25	12.64 ± 0.43	16.55 ± 0.61	38.42 ± 1.21
LP1	20.37 ± 1.19	3.39 ± 0.18	11.69 ± 0.60	15.08 ± 0.72	35.47 ± 1.79
LP2	23.06 ± 1.23	3.98 ± 0.14	12.86 ± 0.69	16.84 ± 0.80	39.90 ± 1.98
LP3	21.32 ± 1.12	4.01 ± 0.23	11.89 ± 0.69	15.91 ± 0.87	37.23 ± 1.83
p-value	NS^3^	NS	NS	NS	NS

^1^C - control diet; LP1 – soybean meal-based diet with reduced crude protein levels (−2%); LP2 – soybean meal-based diet with reduced crude protein levels (−2%) and higher crystalline L-Threonine supplementation; LP3 – diet with reduced crude protein levels (−2%) in which soybean meal partially replaced with swine meat meal as a protein source; ^2^The sum of NH_4_^+^-N and uric acid-N was considered as urinary-N content; ^3^NS – non-significant (*p* > 0.05).

## 4 Discussion

In our study, the dietary LP treatments were applied only from the growing phase. A common starter diet was fed because an early CP reduction would have had minimal impact on the total CP and soybean meal reduction, nitrogen emission, and final performance. In most trials, the reduction in dietary CP by 2% could be achieved without impaired production traits when at least three-phase feeding was used, pelleted diets were fed, and the diets were balanced in at least six limiting AAs (15, 30, 31). In our study, all performance traits of broilers fed LP diets met or exceeded the performance parameters of control birds fed the C diet. The essential AA contents of diets were set according to the ideal protein concept, and the formulation was based on both total and SID AA requirements. Regarding the crystalline essential AA supplementation, not only the four first limiting amino acids (Lys, Met, Thr, and Val) but also L-Arg and L-Ile were used. Broilers fed LP diets had a generally low performance in some studies, which can be explained by the fact that crystalline L-Val, L-Arg, and L-Ile were not used (32, 33). In some studies, when LP diets provided an imbalanced essential AA supply, birds regulated their imbalanced AA intake via hyperphagia, and a significantly higher FI was observed (34, 35). The feeding of LP diets in our study did not lead to a significant increase in FI compared to the C treatment. Furthermore, the intended same SID AA intake in the case of six main essential AAs and the targeted differences of SID Thr and Gly + Ser intake of broilers between the LP and C groups were realized.

The performance of broilers in the LP1 group was similar or even higher (BWG in the finisher phase) compared to those in the C group both in the grower and finisher phases. It means that the extent of decreased Gly supply (Gly_equi_ of 15.65 vs. 13.74 and 14.19 vs. 12.30 in the grower and finisher phases, respectively) while meeting Thr requirements of birds did not impair broiler performance in a significant manner. The explanation for the higher BWG of birds in the finisher phase is not clear, but it can be associated with the increased digestibility of AA in the LP1 diet compared to the C diet. Liu et al. (4) showed that reduced CP feeding generally increases the AA digestibility in the distal jejunum, probably due to the larger ratio of essential crystalline AA found in the reduced protein diets. In the finisher phase, the FCR improvement of broilers in the LP3 group compared to the C group was even better than those in the LP1 group. This result suggests that an increase in the Gly_equi_ of finisher LP diets from 12.3 to 13.55 g/kg is advisable concerning the FCR of broilers. Similar to our results, previous studies have shown that the increased supply of Gly had the most impact on improving FCR (36, 37), possibly by improving nutrient utilization and protein synthesis by enterocyte development and mucin secretion (38). According to the existing literature, the requirement for Gly_equi_ from 0 to 21-day-old broiler chickens is estimated to vary between 11 and 20 g/kg depending on the uric acid formation, Thr supply, Met-to-(Met+Cys) ratio, and choline level (22, 23). Other studies have found an optimum Gly + Ser ranging from 1.8 to 2.3% from 0 to 35 days of age (16, 18, 19). The dietary Gly + Ser level necessary to optimize FCR from 21 to 35 days in LP diets was estimated to be 1.54% at the digestible Thr level of 0.77% (19). The 1.57% calculated Gly + Ser concentration in our finisher LP3 diet, together with the 0.65% SID Thr level, seems to be low, and a further effective increase might be possible. The minimum SID (Gly + Ser)-to-Lys ratios in grower 2 (21–31 days) and finisher phases (31–41 days) are 1.42 and 1.40, respectively, as suggested by Rostagno et al. (39) and confirmed by Mansilla et al. (40). The positive effects of the increased SID (Gly + Ser)-to-Lys ratio in our finisher LP3 diet compared to the LP1 diet (1.30 vs. 1.24) on the FCR of broilers supports a further increase toward the suggested SID (Gly + Ser)-to-Lys ratios.

The relationship between dietary Gly_equi_ and Thr concerning performance traits has been demonstrated (17, 18) and quantified for the phase of 7 to 21 days (22, 23). The decreasing Gly_equi_ impairs the BWG and FCR of broilers in a linear and non-linear manner, respectively, and the negative effect is more pronounced at a lower dietary level of Thr. In the case of FCR, the positive response of birds to the same increase of dietary Thr level is higher at the lower level of Gly_equi_ concentration. Furthermore, the positive effect of a 1 g/kg increase in dietary Thr level on the FCR is higher than the effect of the same increase of Gly_equi_ concentration in the feed. According to the relationship, it is possible to improve FCR by increasing Thr supply while the Gly_equi_ is decreasing in the diet. The treatment LP2 meant similar changes in dietary Thr and Gly_equi_ supplies in comparison with the C treatment in our study, and positive effects on the performance of birds were observed. This positive effect was still a tendency in the grower phase, but it became significant by the end of fattening. The performance improvement of LP2 treatment means that the increase of SID Thr concentration by 0.10% (0.74% vs. 0.84% in the grower and 0.65% vs. 0.74% in the finisher phase) and the increase of SID Thr-to-Lys ratio by 9.0% (63 vs.72% in the grower and 64% vs. 73% in the finisher phase) compared to a control treatment can be beneficial when dietary CP is reduced by 2% and Gly_equi_ by 2 g/kg. The increased supply of birds with Thr in the LP2 treatment group could support the primary functions of Thr for protein synthesis. The enzymatic conversion of Thr to Gly, the so-called replacement effect, may also explain these results (23). In addition, the reduced catabolism of AA other than Thr during increased Thr supply may reduce the need for Gly_equi_ in uric acid formation, which could serve as another Gly-sparing effect (23). Unfortunately, the relationship between dietary Gly_equi_ and Thr concerning performance traits for the whole fattening from hatch to 35 or 42 days of age is not so precisely quantified as it was made for the phase from 7 to 21 days (22). However, similar interactions between Thr and Gly_equi_ may exist until the end of the finisher phase based on our results. Similarly, previous studies focusing on the second half of the fattening confirmed the above-mentioned Thr-Gly interaction, in which the positive performance response of birds to the same increase of dietary Thr level is higher at the lower level of Gly_equi_ concentration. Response of FCR to dietary Thr level was stronger at the lower digestible (Gly + Ser)-to-Lys level (135 vs. 149%) (19). BWG of broilers from 21 to 42 days responded more for Thr when they were fed at 143% compared to 153% digestible (Gly + Ser)-to-Lys ratio level (17). Further studies are needed to quantify the effects of Thr-Gly interaction on broiler performance concerning the whole fattening period.

In the present study, the results of birds in the LP1 treatment are in line with similar previous studies showing no effect of essential AA-supplemented LP diets up to 2% CP reduction on the yield of carcass and valuable carcass parts (30, 40–42). In the study by Mansilla et al. (40), the reductions of SID (Gly + Ser)-to-Lys ratio parallel with the 2% CP reduction compared to the control diet were 5.0, 9.0, and 12.0% in the grower1, grower2 and finisher diets, respectively. The reductions of the SID (Gly + Ser)-to-Lys ratio were higher (15.0% in the grower and 19% in the finisher) in the present experiment, but the carcass weight results were similar in both studies. Only a few results have been published on the effects of Thr––Gly interaction concerning product quality of broilers fed LP diets. Similar to our results observed in the LP2 group, the increase of the digestible Thr concentration from 0.57 to 0.65% while decreasing the Gly + Ser level from 1.65 to 1.55% did not influence the relative carcass weight and breast fillet weight of birds fed an LP diet with 18.2% CP from 21 to 42 days of age (17). An increase of dietary Thr from 9.3 to 10.7 g/kg can even decrease the relative breast weight, which may be due to the toxic effect of increased plasma uric acid and ammonia concentrations (18). In the same study, there was a positive significant linear effect of increasing Gly + Ser concentrations from 18.4 to 22.6 g/kg on the relative breast weight of broilers fed an LP diet containing 19% CP from 0 to 21 days (18).

Our results suggest that the significant negative effects of LP3 treatment on carcass weight and composition could be associated with the starch-to-CP ratio of experimental diets. The content of starch as the main energy provider nutrient typically increases when dietary CP is reduced in isocaloric LP diets (4). In contrast, dietary lipid level usually decreases with the protein level, which was the case in this experiment as well. The starch-to-CP ratio increased in the LP treatments compared to the C treatment, and it was the highest in the LP3 diet containing swine meat meal. The higher starch-to-CP ratio deteriorated the FCR value in the starter phase of our previous experiment in a quadratic manner (43). A similar quadratic relationship was observed between these two factors from 7 to 35 days in two studies (44, 45). In the present study, the higher starch-to-CP ratio of LP diets did not impair the FCR of birds compared to the C diet. In our opinion, however, the highest starch-to-CP ratio in the LP3 diets (2.25 and 2.63 in the grower and finisher phases, respectively) resulted in a significantly lower relative carcass weight in comparison with those in the C diets (1.67 and 2.0 in the grower and finisher phases, respectively). This effect could be seen as a strong tendency in the case of breast meat yield. Based on the digestive dynamics of dietary starch, the absorption of its glucose content has been shown to compete with AA absorption, which may affect the availability of AA for tissue protein accretion (46, 47). However, Hilliar et al. (48, 49) found that the addition of crystalline Gly in an LP diet reduced breast meat yield compared to the control without additional Gly. In this case, the theoretical starch effect as an explanation can be excluded, but the cause is unclear. In contrast to the results of many previous trials (47, 50, 51), feeding the isoenergetic LP1 and LP2 diet did not increase the abdominal fat pad significantly compared to the C diet. However, a slight negative tendency was observed in these two treatments as well, which had a significant negative effect in the LP3 treatment group. The dietary AMEn-to-CP ratio increases while maintaining the dietary AMEn of LP diets constant, and the surplus energy can increase abdominal fat (50, 51). In other studies, the reduction in dietary energy and CP while maintaining the same AMEn-to-CP ratio successfully prevented the accumulation of abdominal fat, but the growth performance of broilers was suppressed (52, 53). The AMEn-to-CP ratio of LP diets was nearly the same, but only the LP3 treatment resulted in a significant increase in the abdominal fat pad ratio. The use of synthetic Gly supplementation can reduce the fatness of broilers fed LP diets (37, 54, 55). According to studies with poultry, rats, and swine, increasing dietary Gly or betaine (trimethylglycine) has been demonstrated to stimulate lipid oxidation and reduce plasma concentrations of triglycerides and fat deposition (56–58). In contrast, the use of swine meat meals to increase the Gly + Ser concentration of LP3 diets led to opposite results in the present study. We assume that the higher starch-to-CP ratio in the LP3 diet compared to the LP1 and LP2 diets was associated with the significantly increased abdominal fat ratio. If the tissue protein accretion was decreased in the LP3 group due to the high starch-to-CP ratio, as assumed based on the carcass weight result, the surplus energy formed could lead to an accumulation of abdominal fat pad in a significant manner. Another possible explanation is the elevated hepatic acetyl-CoA concentration derived from relatively high dietary starch levels in birds offered LP diets (4). Acetyl-CoA can serve as a precursor for fatty acid synthesis and influence the activity of numerous enzymes (4). These negative consequences of LP diets supplemented with 6 and 4% swine meat meal on carcass weight and abdominal fat pad need more focused investigations. In addition to the dietary balance of AA, the digestive dynamics of main nutrients, especially starch, lipid, and protein, should be considered in the further development of LP diets.

As we know, the effects of dietary Thr-Gly interaction on the meat quality of broilers fed LP diets have not been investigated. The pH and drip loss of breast meat in the present experiment did not show significant changes due to dietary LP treatments with different Thr and Gly_equi_ supplies. The drip loss is one of the parameters that is associated with the water-holding capacity of meat and influences its sensory and technological quality. The negative relationship between drip loss and ultimate pH in poultry meat is well known (52, 59). The feeding of LP diets can result in higher ultimate pH and decreased drip loss (43, 60). The higher drip loss of the meat proved to be more acidic with a higher level of glucose, glycogen, and glycolytic potentials (52, 53, 59). The post-mortem breakdown of the glycogen accumulated in the muscle tissues is responsible for the proper acidity of the meat after slaughter. If an adequate amount of glycogen is not available, the pH of the meat becomes less acidic, and the water-holding capacity of meat is higher. The drip loss of meat could be associated with excess AAs (60). After the deamination of the not utilized AAs, the carbon chain is used by the muscle tissue for the synthesis of various carbohydrates, such as glycogen. This suggests that our experimental diets provided a similar balanced AA profile without or with a similar amount of excess AA.

The dietary N retention efficiency of broilers measured in our experiment was improved by 3–7% with LP diets compared to the C diet, and this improvement is in line with the previously published results (2–13%) of LP diets providing an adequate essential AA supply (40, 61, 62). However, the N retention efficiency of broilers has not been reported by the dose–response studies with Thr-Gly supplemented LP diets (17–19, 21). The mean increase of the efficiency of N retention was +2.63%/CP percentage point, which is a little lower than the corresponding value of +3.2% published by Belloir et al. (15). The improvements were strong tendencies in the LP1 and LP2 groups compared to the C group, and it was a significant difference between LP3 and C treatments. According to our result, the increased level of Gly_equi_ in LP diets with 2% CP reduction can be recommended to increase N retention efficiency, as it was advisable to increase the FCR of finishing birds as well. Extremely advantageous efficiency values higher than 70% were measured in the LP1 and LP3 treatments, which have also been reported by other research groups (14, 15). The increasing N utilization efficiency decreases the dietary Gly_equi_ needed for uric acid synthesis based on model calculations (63, 64). The estimation shows that an N utilization efficiency higher than 70% requires only less than 10 g/kg Gly_equi_ for uric acid production. Most of the ammonia released from poultry manure originates from the breakdown of uric acid (65). The feeding of LP diets can decrease the uric acid level of blood plasma (21) and the concentrations of total and urinary N and uric acid in broiler excreta (15, 31, 43). However, the results of the present experiment failed to confirm the advantageous results of previous studies associated with N emission. The uric acid concentration of excreta in broilers fed the control C diet was already quite low (12.54 mg/g dry matter), and maybe a further decrease was not possible by feeding LP diets. In our previous two experiments, the LP diets reduced the uric acid levels in the excreta when higher uric acid levels than in the present trial were measured in the excreta of broilers fed normal CP diets (17.65 and 15.30 mg/g dry matter) (31, 43).

In addition to the performance traits, the economic effectiveness of LP diets depends on the actual prices of raw materials, especially soybean meal and crystalline AA supplements. According to the present Hungarian prices, the cost of the experimental diets based on the ingredients only were 364, 362, 363, and 352 EUR per ton in the case of the grower, and 346, 343, 344, and 339 EUR per ton in the case of finisher diets of C, LP1, LP2 and LP3 treatments, respectively. All the LP diets had lower prices than the C diet, and assuming similar performance of birds fed C and LP diets, they can contribute to higher profitability of broiler production.

## 5 Conclusion

According to the results of this experiment, the increased SID Thr-to-Lys ratio and Gly_equi_ of LP diets with 2% lower CP content than adequate may have positive effects on broiler performance and nitrogen retention efficiency. The dietary SID Thr-to-Lys ratio of LP diets higher than recommended can improve the final BW, BWG, and FCR of birds. Swine meat meal as a source of Gly can be used to increase Gly_equi_ in practical LP diets in the EU, which can lead to a more advantageous FCR in the finisher phase and higher N retention efficiency. However, the LP diets containing swine meat meal may have a high starch-to-CP ratio, which can contribute to a decreased relative carcass weight and an increased abdominal fat pad ratio of broilers at market age.

## Data availability statement

The raw data supporting the conclusions of this article will be made available by the authors, without undue reservation.

## Ethics statement

The animal study was approved by Animal Welfare Committee, Hungarian University of Agriculture and Life Sciences, Georgikon Campus, under the license number MÁB-3/2022. The study was conducted in accordance with the local legislation and institutional requirements.

## Author contributions

PS: Data curation, Formal analysis, Methodology, Project administration, Writing – original draft. BH: Data curation, Formal analysis, Methodology, Project administration, Writing – original draft. NS: Data curation, Formal analysis, Methodology, Project administration, Writing – original draft. KD: Resources, Supervision, Validation, Writing – original draft, Writing – review & editing. LP: Conceptualization, Investigation, Methodology, Project administration, Resources, Supervision, Validation, Writing – original draft, Writing – review & editing.
